# Incidence of Precipitated Withdrawal During a Multisite Emergency Department–Initiated Buprenorphine Clinical Trial in the Era of Fentanyl

**DOI:** 10.1001/jamanetworkopen.2023.6108

**Published:** 2023-03-30

**Authors:** Gail D’Onofrio, Kathryn F. Hawk, Jeanmarie Perrone, Sharon L. Walsh, Michelle R. Lofwall, David A. Fiellin, Andrew Herring

**Affiliations:** 1 Department of Emergency Medicine, Yale School of Medicine, New Haven, Connecticut; 2 Department of Internal Medicine, Yale School of Medicine, New Haven, Connecticut; 3 School of Public Health, Yale School of Medicine, New Haven, Connecticut; 4 Department of Emergency Medicine, Perelman School of Medicine at the University of Pennsylvania, Philadelphia; 5 Center on Drug and Alcohol Research, University of Kentucky College of Medicine, Lexington; 6 Department of Emergency Medicine, Highland Hospital Oakland, Oakland, California

## Abstract

This cohort study examines the incidence of precipitated withdrawal comparing traditional sublingual buprenorphine with a 7-day extended-release injectable initiated in the emergency department (ED).

## Introduction

Buprenorphine treatment is associated with decreased mortality and morbidity,^1^ yet the treatment gap remains wide. Emergency departments (EDs) offer an effective, low-barrier setting in which to initiate buprenorphine.^2^ Retrospective case series^3^ have raised concerns about increased incidence of precipitated withdrawal (PW) when buprenorphine is initiated in persons using fentanyl, a high-potency μ-opioid agonist with high affinity and slow dissociation from the μ receptor. With long-term use, its high lipophilicity leads to bioaccumulation and prolonged metabolite excretion. As confidence in standard buprenorphine inductions has eroded, alternative strategies, such as low-dose buprenorphine, have emerged, often prompting continued use of illicit opioids. Thus, there is a need for high-quality evidence from prospective studies using uniform surveillance and operational definitions of PW. We report the incidence of PW as part of an ongoing randomized clinical trial^4^ comparing traditional sublingual buprenorphine with CAM2038, a 7-day extended-release injectable form of buprenorphine, conducted in sites with high prevalence of fentanyl.

## Methods

This observational cohort study using data from an ongoing clinical trial^4^ included patients aged 18 years or older with moderate-to-severe opioid use disorder, opioid-positive and methadone-negative urine tests, and a Clinical Opiate Withdrawal Scale (COWS) score of 4 or higher. Pregnant or admitted patients were excluded. Twenty-eight geographically diverse EDs participated from June 30, 2020, to October 26, 2022. Patients were randomized to standard sublingual buprenorphine inductions or extended-release buprenorphine and were observed for 2 hours. PW was defined^5^ a priori and was considered when a marked escalation in objective COWS scores (score ≥5) occurred, requiring additional buprenorphine and ancillary medications, often within 2 hours of buprenorphine administration. Suspected PW cases were documented prospectively and adjudicated by expert consultants (S.L.W. and M.R.L.). This study was reported according to the Strengthening the Reporting of Observational Studies in Epidemiology (STROBE) reporting guideline for cohort studies and was approved by the WCG institutional review board. Participants provided written consent.

Patients with a COWS score of 8 or higher received 8 mg of sublingual buprenorphine in the ED and were discharged with a prescription for 16 mg/day. Individuals with COWS scores of 4 to 7 received uniform instructions for unobserved induction, up to 12 mg the first day, and then 16 mg/day. In the extended-release buprenorphine group, on the basis of dose equivalency to 16 mg of sublingual buprenorphine, patients received 24 mg of injectable CAM2038 in the ED during the index visit and follow-up.^3^ Data analysis was performed with SAS statistical software version 9.4 (SAS Institute).

## Results

Among 1200 enrolled patients (800 men [66.7%]; mean [SD] age, 38.4 [12.0] years), there were 9 cases of PW, or 0.76% (95% CI, 0.35%-1.43%) of the overall sample. Patient characteristics (total and PW) are presented in Table 1. The PW cases were enrolled from diverse locations; 5 received sublingual buprenorphine and 4 received extended-release buprenorphine. Detailed data from the PW cases are reported in Table 2. All patients had urine tests positive for fentanyl, 7 with multiple drugs. Routes of use, changes in baseline and peak COWS scores, and time elapsed from buprenorphine administration to PW varied. Time since last use ranged from 8 to more than 24 hours. All patients experiencing PW were discharged after symptoms resolved, with 1 self-directed discharge. Follow-up rates at 7 days after the ED visit were 86%.

**Table 1.** Clinical Characteristics

Clinical characteristics	Participants, No. (%)	
	Total (N = 1200)	Precipitated withdrawal (n = 9)
Sex		
Male	800 (66.7)	6 (66.7)
Female	400 (33.3)	3 (33.3)
Age, mean (SD), y	38.4 (12.0)	38.3 (13.6)
Race		
American Indian or Alaskan Native	26 (2.2)	1 (11.1)
Asian	9 (0.8)	0
Black or African American	361 (30.1)	4 (44.4)
Multiracial	22 (1.8)	2 (22.2)
White	674 (56.2)	2 (22.2)
Other, unknown, or refused^a^	108 (9.0)	0
Ethnicity, Hispanic or Latino	168 (14.0)	0
Unstable housing past 12 mo^b^	593 (50.51)	4 (44.4)
Currently living in unstable housing^b^	408 (34.7)	4 (44.4)
Urine point of care testing		
Opioids plus any other drug^c^	1003 (83.6)	6 (66.7)
Fentanyl plus any other drug	834 (69.5)	7 (77.8)
Fentanyl plus any other opioid	533 (44.4)	2 (22.2)
Fentanyl plus no other opioid	364 (30.3)	7 (77.8)
Fentanyl only (no other drug)	63 (5.3)	2 (22.2)
Opioids plus any stimulant (cocaine, amphetamine, or methamphetamine)^c^	722 (60.2)	6 (66.7)
Opioids plus amphetamine or methamphetamine^c^	451 (37.6)	0
Amphetamine	391 (32.6)	0
Barbiturate	11 (0.9)	0
Benzodiazepines	198 (16.5)	0
Buprenorphine	442 (36.8)	0
Cocaine	401 (33.4)	6 (66.7)
Marijuana	551 (45.9)	4 (44.4)
3,4-Methyl​enedioxy​methamphetamine	121 (10.1)	0
Methamphetamine	410 (34.2)	0
Opiates	498 (41.5)	2 (22.2)
Oxycodone	81 (6.8)	0
Phencyclidine	21 (1.8)	0
Route of use		
Oral	119 (9.9)	0
Nasal	395 (32.9)	3 (33.3)
Injection	328 (27.3)	3 (33.3)
Smoking	197 (16.4)	3 (33.3)
Multiple noninjection	40 (3.3)	0
Other, not applicable, or unknown	121 (10.0)	0
Severity of use (7-d past use), mean (SD), d	5.31 (2.30)	6.78 (0.40)

^a^ Other race refers to unknown or refused to specify.

^b^ Unstable housing is defined as spending at least 1 night in a shelter for the homeless; on the street or in a public place; in a welfare hotel; doubled up in someone else’s house or apartment; or in an emergency, temporary, transitional, or halfway house.

^c^ Opioids includes buprenorphine, fentanyl, opiates, or oxycodone.

**Table 2.** Detailed Data From PW Cases^a^

Enrollment date	Location	Age, decade	Race	Gender	Severity of use, d/wk	Last use, h	Route	Urine drug testing	BUP type	COWS scores, baseline/peak	Time elapsed, min^b^	Disposition	ED LOS
December 2020	Northeast	50s	Black	Woman	7	16	Injection	Opiates and fentanyl	SL	13/19	20	Discharged	6 h 40 min
January 2021	West	20s	White	Woman	7	8	Smoking	Fentanyl	XR	15/23	25	Discharged	2 h 50 min
February 2021	Northeast	40s	White	Man	7	8	Nasal	Fentanyl	XR	12/20	114	Observation^c^	7 h 50 min
												Discharged	
April 2021	Midwest	60s	Black	Woman	7	24	Nasal	Cocaine, opiates, marijuana, fentanyl	XR	8/16	60	Against medical advice	1 h 41 min
May 2021	Northeast	30s	Multiracial	Man	6	>24	Injection	Cocaine, marijuana, fentanyl	SL	17/23	54	Discharged	7 h 24 min
August 2021	South	30s	Multiracial	Man	6	24	Smoking	Cocaine, fentanyl	SL	13/32	55	Observation^c^	22 h 39 min
												Discharged	
September 2021	Midwest	40s	Black	Man	7	12	Nasal	Cocaine, marijuana, fentanyl	XR	13/20	30	Discharged	8 h 50 min
November 2021	Midwest	20s	American Indian/Alaskan Native	Man	7	16	Smoking	Cocaine, marijuana, fentanyl	SL	10/22	82	Discharged	8 h 43 min
December 2021	South	20s	Black	Man	7	15	Injection	Cocaine, fentanyl	SL	29/>30^d^	116	Observation^c^	20 h 0 min
												Discharged	

Abbreviations: BUP, buprenorphine; COWS, Clinical Opiate Withdrawal Scale; ED, emergency department; LOS, length of stay; PW, precipitated withdrawal; SL, sublingual; XR, extended-release injectable.

^a^ Rates of PW by region were as follows: Northeast (10 sites), 3 of 313 participants (0.95%); West (6 sites), 1 of 423 participants (0.24%); Midwest (6 sites), 3 of 207 participants (1.44%); South (6 sites), 2 of 257 participants (0.78%); and total, 9 of 1200 participants (0.76%).

^b^ Time elapsed from BUP administration to maximum COWS score.

^c^ Patient was placed in ED observation status and then discharged.

^d^ COWS score improved to 15 then increased (exact score unobtainable due to patient’s condition).

## Discussion

This cohort study used data from the first, to our knowledge, prospective trial^4^ using uniform surveillance, operational definitions, and adjudicated outcomes to document buprenorphine-induced PW in persons using fentanyl. Despite high fentanyl prevalence, the incidence of PW in this multisite trial^4^ of ED-initiated buprenorphine was less than 1%, similar to reported rates among persons using heroin or prescription opioids.^6^ All 9 patients with PW used fentanyl, most without PW also used fentanyl, and no factors suggest a specific phenotype for PW. The discordance between our findings and those of retrospective studies is striking.

Limitations include possible undetected fentanyl analogues or nitazenes leading to PW. We may have missed PW after discharge, although follow-up rates at 7 days after the ED visit were 86% and likely would be captured as adverse events.

In this geographically diverse observational cohort, buprenorphine induction in the ED remained safe and effective, even with fentanyl present. Continued access to buprenorphine for opioid use disorder treatment is essential given the ongoing overdose crisis.

## Notes

Supplement.
Data Sharing Statement
