# Supplementary material for: Incidence of Precipitated Withdrawal During a Multisite Emergency Department–Initiated Buprenorphine Clinical Trial in the Era of Fentanyl
Source: JAMA Netw Open. 2023 Mar 30;6(3):e236108. doi: 10.1001/jamanetworkopen.2023.6108 (PMC10064247; doi:10.1001/jamanetworkopen.2023.6108)
Supplement: Supplement. — Data Sharing Statement [file jamanetwopen-e236108-s001.pdf]

Data Sharing Statement

D'Onofrio. Incidence of Precipitated Withdrawal During a Multisite Emergency Department-Initiated Buprenorphine Clinical Trial in the Era of Fentanyl. *JAMA Netw Open*. Published March 30, 2023. doi:10.1001/jamanetworkopen.2023.6108

Data
Data available: No

Additional Information
Explanation for why data not available: This is a cohort study of individuals enrolled in a randomized clinical trial testing two formulations of buprenorphine, sublingual and a 7-day extended release injectable to determine their effectiveness in engagement in treatment at 7 days. We cannot release patient level data until the entire sample of 2000 is complete. However, this extraordinary finding should be published soon. All data will eventually be available with the publication of the clinical trial
